# Older adults' preferences for and actual situations of artificial hydration and nutrition in end‐of‐life care: An 11‐year follow‐up study in a care home

**DOI:** 10.1111/ggi.14419

**Published:** 2022-06-18

**Authors:** Taizo Wada, Yasuko Ishimoto, Kiichi Hirayama, Emiko Kato, Mai Tatsuno, Michiko Fujisawa, Yumi Kimura, Yoriko Kasahara, Eriko Fukutomi, Hissei Imai, Masahiro Nakatsuka, Mitsuhiro Nose, Masanori Iwasaki, Satoko Kakuta, Mayumi Hirosaki, Kiyohito Okumiya, Kozo Matsubayashi, Ryota Sakamoto

**Affiliations:** ^1^ Center for Southeast Asian Studies Kyoto University Kyoto Japan; ^2^ Osaka Home Healthcare Clinic Suita Japan; ^3^ Department of Health and Sports Science, Faculty of Health Science and Technology Kawasaki University of Medical Welfare Kurashiki Japan; ^4^ Department of Field Medicine Kyoto University Graduate School of Medicine Kyoto Japan; ^5^ Graduate School of Human Sciences Osaka University Suita Japan; ^6^ Division of Human Health Sciences, Department of Fundamental Nursing Kyoto University Graduate School of Medicine Kyoto Japan; ^7^ Yoshino Town Office Yoshino Japan; ^8^ Department of Health Promotion and Human Behavior Kyoto University School of Public Health Kyoto Japan; ^9^ Gifu Shotoku Gakuen University Gifu Japan; ^10^ Tokyo Metropolitan Institute of Gerontology Tokyo Japan; ^11^ Division of Community Oral Health Development Kyushu Dental University Kitakyushu Japan

**Keywords:** advance care planning, advance directives, artificial hydration and nutrition, end‐of‐life care, preferences

## Abstract

**Aim:**

To clarify older adults' preferences for and actual situations of artificial hydration and nutrition (AHN) in end‐of‐life care in a care home.

**Methods:**

Participants were residents of a care home who had completed advance directives regarding preferred methods of AHN from 2009 to 2018. Advance directives alone were available from April 2009 to June 2016 (Wave 1), and advance care planning for AHN including advance directives was introduced in July 2016 (Wave 2). AHN preferences included (i) intensive methods (percutaneous endoscopic gastrostomy, nasogastric tube feeding and total parenteral nutrition), (ii) drip infusion, and (iii) oral intake only. Participants were followed until the end of 2020, and we checked whether decisions about AHN were based on older adults' preferences.

**Results:**

In total, 272 participants had completed advance directives. Most participants preferred “oral intake only” (59.5%), followed by drip infusion (32.0%) and intensive methods (8.5%) in advance directives. Ninety of the 272 participants completed advance directives twice; 83.3% did not change their AHN preferences from Wave 1 to Wave 2. By the end of 2020, 93 of the 272 participants died in the care home. AHN was provided according to older adults' preferences in 48.9% (oral intake only), in 51.4% (drip infusion) and in 55.6% (intensive methods) of cases respectively.

**Conclusions:**

Most participants preferred oral intake only, and their preferences were reflected in decisions about actual situations of AHN in end‐of‐life care. To prepare for advanced dementia and senility, early advance care planning for AHN should be promoted. **Geriatr Gerontol Int 2022; 22: 581–587**.

## Graphical abstract

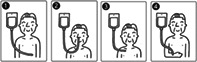

Advance care planning for artificial hydration and nutrition (AHN) have been introduced in a care home. Most participants preferred oral intake only, (59.5%), followed by drip infusion (32.0%) and intensive methods (percutaneous endoscopic gastrostomy, nasogastric tube feeding, and total parenteral nutrition) (8.5%).Their preferences were reflected in decisions about AHN in end‐of‐life care.

## Introduction

In Japan, the rate of dementia has increased with the rate of aging, and senility has been the third leading cause of death since 2018.^1^ In one study, the prevalence of dementia in people ≥65 years was 15.7% in 2014 (*n* = 1537) but the estimated future prevalence of dementia for the entire country projected to exceed 25% by 2035.^2^ Artificial hydration and nutrition (AHN) for dysphagia due to advanced dementia or senility has been controversial in the area of elderly end‐of‐life care.^3^ Although tube feeding has been associated with adverse effects such as aspiration pneumonia and malnutrition,^4^, ^5^ enteral nutrition can prolong the survival of patients with dementia.^6^, ^7^ While the use of percutaneous endoscopic gastrostomy (PEG) is decreasing in Japan,^8^ PEG as well as nasogastric (NG) tube placement and total parenteral nutrition (TPN) are still often performed in patients with advanced dementia when their preferences are unknown. As it is usually the case that older patients lose their decision‐making capacity before the need to make decisions about AHN arises, advance care planning (ACP)^9,^ ^10^ should be initiated early on to ensure that patient values and intentions are respected. In addition, this will avoid ethical dilemmas for family caregivers and medical staff. Our preliminary study indicated that 4–5% of community‐dwelling older people prefer PEG and NG tube feeding or TPN when they become unable to achieve sufficient nutrition orally, 42.5% prefer drip intravenous infusion and 50.3% prefer oral intake only.^11^

Caregivers often experience ethical dilemmas when their loved ones choose to forgo AHN at the end of life. Although little scientific evidence exists on the effectiveness of PEG for prolonging patient survival,^4^, ^5^ PEG could improve the emotional state of family caregivers of patients with advanced dementia, as some are willing to provide care at home and/or find it difficult to accept the idea of forgoing AHN. In 2012, the Japanese Geriatric Society issued guidelines for decision‐making processes in elderly care focusing on the introduction of AHN.^12^ ^,^ ^13^ The philosophy behind the guidelines is that ACP is vital for initiating tube feeding in end‐of‐life situations.

Life in Kyoto is a care home in Kyoto, Japan. Since 2009, preferences of residents for AHN in end‐of‐life care have been included in the original advance directives (AD) document at Life in Kyoto. According to Monturo and Strumpf, only a small percentage (8.8%) (*n* = 57) of nursing home residents received tube feeding at the end of life, and of these, only one case was consistent with AD.^14^ However, only a few prospective studies have investigated whether decisions about AHN are based on patient AD.

The purpose of this study was to clarify the feeding methods preferred by older people living in a care home when they develop dysphagia due to aging or diseases, and compare their preferences with the actual situations of AHN in end‐of‐life care.

## Methods

### Setting and participants

This study was performed in a care home, Life in Kyoto, which has been providing residential care, nursing care and palliative care to older residents since 1986. This 308‐unit care home has 172 employees, of whom 28 are qualified nurses. Between 2009 and 2018, in total, 529 older adults lived in this care home. The number of residents at baseline (April 1, 2009) was 319 (82 men and 237 women, mean age ± SD, 82.6 ± 6.3). The care home differs from regular nursing homes with regard to admission requirements, level of care, education years of resident and resident autonomy. Residents must pay a relatively large sum of money for the right to reside in the care home and pay monthly fees to access common facilities. Median education years of residents were 14 (interquartile range, 12–16) and many of the residents had a university‐level educational background.

Since 2009, AD documents, which include AHN preferences in end‐of‐life care, the do‐not‐resuscitate order and the desired place to die have been made available to all residents of Life in Kyoto to complete before they lose their decision‐making capacity. With an option to choose oral intake only, residents are asked to answer the question, “If you cannot take meals orally due to end‐stage dementia, senility, or any other diseases with no possibility to improve, which feeding method do you prefer?” Five choices are provided, including (i) PEG, (ii) NG tube feeding, (iii) TPN, (iv) drip infusion through peripheral vein (DIV) and (v) oral intake only. AD documents are always available for residents who have the capacity to express their own preferences as they wish. When a resident completes the AD, a family member or advocate approves the document, which is then checked by the chief manager of Life in Kyoto. In this study, we set the following inclusion criteria: residents aged ≥65 years with decision‐making capacity who completed the AD and agreed to participate in this study. Residents who had lost their decision‐making capacity, or who had been introduced PEG, NG tube feeding or TPN at the time of AD were excluded. All AD documents were checked by a qualified nurse and approved by the chief manager.

### Comprehensive geriatric assessment

Baseline data from comprehensive geriatric assessment (CGA) were checked within the year of the latest AD approval. Basic activities of daily living were assessed using a seven‐item questionnaire on (i) walking, (ii) ascending and descending stairs, (iii) feeding, (iv) dressing, (v) using the toilet, (vi) bathing and (vii) grooming. These items were scored from 0 (completely dependent) to 3 (completely independent), with a full score of 21 indicating complete independence.^15^ Higher‐level daily activities were assessed using the Tokyo Metropolitan Institute of Gerontology Index of Competence (TMIG‐IC) questionnaire on a scale of 0 (completely dependent) to 13 (completely independent).^16^ The assessment consisted of the following three components: instrumental self‐maintenance (instrumental activities of daily living, five items; score range: 0–5), intellectual activity (four items; score range: 0–4) and social role (four items; score range: 0–4), with higher scores indicating more independence. Cognitive function was assessed by the Mini‐Mental Scale examination,^17^ if participants agreed. For depression screening, the 15‐item Geriatric Depression Scale (GDS‐15) was used.^18^ Quality of life (QOL) was assessed using a 100‐mm visual analog scale (worst QOL being on the left end of the scale, best on the right) for the subjective sense of health and happiness.^19^, ^20^

### Registration period

The registration period was divided into “AD only” and “AD after ACP conversation,” as follows.

#### From April 2009 to June 2016 (Wave 1: advance directive only)

Each of the AHN methods was briefly explained once a year in a group session as part of a series of lectures on aging gracefully. Participants responded to the question regarding AHN preferences based on their own perception and knowledge.

#### From July 2016 to December 2018 (Wave 2: advance directive after advance care planning conversation)

**Figure 1:**
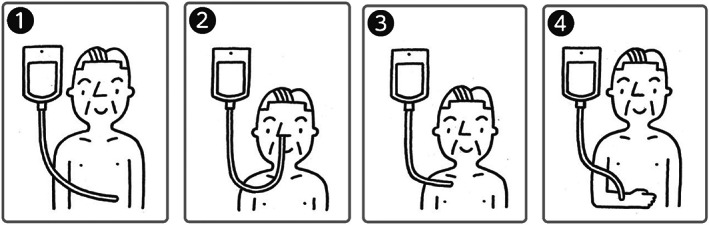
Feeding methods. (1) Percutaneous endoscopic gastrostomy. A hole is opened in the stomach via endoscopic surgery. Advantages: it is an excellent nutritional method, as nutrients are absorbed from the intestinal tract. Less risk of infection than central thoracic parenteral nutrition. If semi‐solid nutrition is used, the caregiver can finish the injection in about 5–10 min. If organ function is normal, it is possible to extend the life of patients by several years. Drawbacks: there is a risk of aspiration pneumonia due to regurgitation of nutritional supplements and chronic misalignment. At first, a minor operation is performed with a gastrocamera. The gastrostomy button needs to be replaced approximately every 6 months. It may be difficult to secure a caregiver because it is often possible to extend the patients' life for several years. (2) Nasogastric tube feeding. A tube is inserted through the nose into the stomach. Advantages: it is an excellent nutritional method, as nutrients are absorbed from the intestinal tract. Less risk of infection than central thoracic parenteral nutrition. No surgery is required. Drawbacks: as semi‐solid nutrition cannot be used, it is necessary to infuse liquid food over 1–2 h. Because of the use of thin and long tubes, they may become obstructed. Tubes need to be replaced every 2–4 weeks, and there is considerable discomfort during insertion each time. There is a risk of aspiration pneumonia due to regurgitation of nutritional supplements and chronic misalignment. The tubes may be accidentally removed. (3) Total parenteral nutrition through a central venous catheter (requires a minor surgery). Advantages: in addition to hydration, patients can be fed sufficient calories. Drawbacks: there is a risk of local infection, and sepsis can occasionally occur. The catheter may be accidentally pulled out. Minor surgery is required. Reoperation is required in the case of obstruction. (4) Drip infusion through peripheral intravenous catheter (water and limited nutrition). Advantages: patients can be easily rehydrated. Subcutaneous infusion is possible even when it is difficult to secure blood vessels. Drawbacks: it is not possible to supply the nutrition necessary for life support. The catheter may be accidentally pulled out. Intravenous drip may leak. Edema may become serious. (5) Oral intake only. Advantages: patients can live naturally according to their desire to eat by mouth. The occurrence of edema is low because water is taken within the range of motivation and swallowing function. Drawbacks: there is a risk of aspiration pneumonia if the caregiver actively brings food to the mouth or due to poor oral hygiene. The risk of aspiration is particularly high if the patient is using sleeping pills or sedatives. Inability to supply the nutrients and water needed to sustain life.

An illustration of each AHN method with a detailed explanation was attached to AD documents (Fig. 1). Participants were encouraged to discuss their choices with staff. During the annual CGA check‐up, medical doctors explained the benefits and drawbacks of each AHN.

If the participant completed the AD document twice or more, the version that was approved the latest was used in this study, with the start date defined as the date of approval by the chief manager.

All participants were followed until December 31, 2020. If participants died in Life in Kyoto, the cause of death and the actual situation regarding AHN in their end‐of‐life care were checked by nursing staff.

### Statistical analysis

The main outcome was the actual status of AHN during end‐of‐life care. Participants with preferences for PEG, NG tube feeding and TPN were classified into the intensive method group. One‐way ANOVA was used to compare continuous variables, and the *post‐hoc* Bonferroni test was used to determine if there were significant differences between groups. The chi‐squared test was used to compare categorical values. The Kaplan–Meier method was used to assess cumulative survival in the three AHN groups, and the log‐rank test was used to compare differences between the groups. All analyses were performed using STATA 14.2 software (Stata Corp. LLC, College Station, TX, USA), with statistical significance set at *P* < 0.05.

### Ethical approval

This study complied with the Declaration of Helsinki and was approved by the Ethics Committee of Kyoto University Graduate School and Faculty of Medicine (E‐18, R2039). All participants provided written informed consent.

## Results

In total, 272 participants (64 men and 208 women; mean age ± SD, 85.8 ± 7.5 years) completed AD at least once by the end of 2018. The participation rate was 51.4% (*n* = 529). Most participants preferred oral intake only (*n* = 162, 59.6%), followed by DIV (*n* = 87, 32.0%), PEG (*n* = 11, 4.0%), NG tube feeding (*n* = 8, 2.9%) and TPN (*n* = 4, 1.5%) (Table 1). During Wave 1 and Wave 2, 201 and 161 participants completed AD, respectively. Among these, 90 completed AD twice as ACP was introduced during Wave 2, of whom 75 (83.3%) chose the same AHN preference, seven (7.8%) changed to a more aggressive option (e.g., from DIV to PEG), and eight (8.9%) changed to a less aggressive option (e.g., from PEG to oral intake only).

**Table 1:** Preferences of artificial hydration and nutrition methods in each wave

	Wave1 (*n*)	%	Wave2 (*n*)	%	Latest AD (*n*)	%
PEG	8	4.0	4	2.5	11	4
NG tube	6	3.0	3	1.9	8	2.9
TPN	4	2.0	1	0.6	4	1.5
DIV	66	32.8	40	24.8	87	32
Oral	117	58.2	113	70.2	162	59.6
Total	201	100.0	161	100.0	272	100

Wave 1: April 2009–June 2016.

Wave 2: July 2016–December 2018.

AD, advance directive; DIV, drip infusion through peripheral vein; NG, nasogastric; PEG, percutaneous endoscopic gastrostomy; TPN, total parenteral nutrition.

CGA data from the year of the latest AD approval were compared between the intensive method group, DIV group and oral intake only group (Table 2). There were no significant differences in mean age, proportion of participants who lived alone, MMSE, GDS‐15, and QOL among the three groups. The oral intake only group had higher ADL compared with the DIV and intensive method groups (*P* < 0.01 and *P* < 0.05, respectively). The proportion of female participants was the highest in the DIV group. Scores of TMIG‐IC, instrumental activities of daily living and intellectual activities were lower in the DIV group compared with the oral intake only group, but did not differ significantly between the DIV and intensive method groups.

**Table 2:** Baseline characteristics of 272 nursing home resident at completion of latest advance directives with preferences of artificial hydration and nutrition methods

		All	Intensive method (PEG/NG/TPN)	DIV	Oral	*P*	
*n* (%)		272	23 (8.5)	87 (29.9)	162 (59.6)		
Age (years), mean ± SD		85.8 ± 7.5	84.6 ± 6.4	86.4 ± 5.9	85.6 ± 8.4	0.53	
Female, %		76.4	63.6	85.1	73.5	0.04	
Living alone, %		63.0	75.0	60.7	63.6	0.85	
BADL (0–21), mean ± SD		16.6 ± 6.1	14.2 ± 7.7*	14.5 ± 7.6**	17.9 ± 4.6	<0.001	
TMIG‐IC (0–13), mean ± SD		8.4 ± 4.7	7.5 ± 6.1	7.0 ± 5.1*	9.1 ± 4.2	0.02	
IADL (0–5), mean ± SD		3.2 ± 2.1	3.0 ± 2.3	2.5 ± 2.2**	3.6 ± 1.9	0.007	
Intellectual activity (0–4), mean ± SD		2.9 ± 1.5	2.5 ± 1.8	2.5 ± 1.6*	3.1 ± 1.4	0.02	
Social role(0–4), mean ± SD		2.2 ± 1.5	2.1 ± 1.8	2.0 ± 1.5	2.3 ± 1.4	0.3	
MMSE (*N* = 82), mean ± SD		25.1 ± 6.1	26.7 ± 5.8	24.5 ± 5.1	25.4 ± 6.5	0.76	
GDS‐15 (0–15), mean ± SD		4.4 ± 3.5	4.6 ± 4.2	4.4 ± 3.4	4.4 ± 3.4	0.98	
Subjective sense of health(0–100), mean ± SD		62.2 ± 21.1	64.8 ± 21.0	60.4 ± 18.4	62.7 ± 22.4	0.74	
Subjective sense of happiness (0–100), mean ± SD		76.5 ± 20.0	70.9 ± 28.2	74.2 ± 21.0	78.2 ± 18.2	0.25	

*P* value: ANOVA for continuous variable, chi squared for categorical variable.

Bonferroni *post hoc* test: oral intake vs. intravenous drip infusion, oral intake vs. TPN or PEG *P < 0.05, **P < 0.01.

BADL, basic activities of daily living; DIV, drip infusion through peripheral vein; GDS‐15, 15‐item geriatric depression scale; IADL, instrumental self‐maintenance; NG, nasogastric; PEG, percutaneous endoscopic gastrostomy; TMIG‐IC, Tokyo Metropolitan Institute of Gerontology Index of Competence; TPN, total parenteral nutrition.

**Figure 2:**
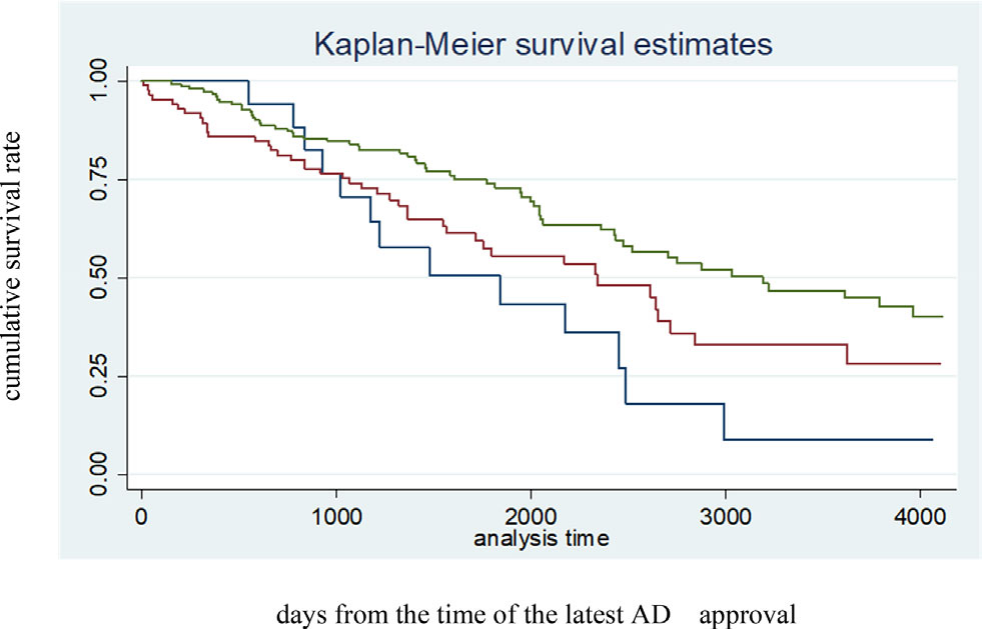
Kaplan–Meier survival estimates for the intensive method group, drip intravenous infusion group and oral intake only group. Green: oral intake only (*N* = 162); Red: drip infusion through peripheral intravenous catheter (*N* = 87); Blue: intensive methods = percutaneous endoscopic gastrostomy + nasogastric tube feeding + total parenteral nutrition through a central venous catheter (*N* = 23). Log‐rank test *P* = 0.09. AD, advance directives.

Among 120 of the 272 participants who died before the end of 2020, 93 died in Life in Kyoto. Senility (56.8%) was the most common cause of death among the 93 participants, followed by cancer (12.6%), pneumonia (9.5%), stroke (3.2%) and others (17.9%). Figure 2 shows Kaplan–Meier survival estimates from the date of latest AD approval. No significant difference was observed among the three groups (*P* = 0.09). Table 3 summarizes AHN preferences at the time of the latest ACP and actual AHN provided during end‐of‐life care. Older adults' preferences were reflected in decisions about AHN in 48.9% (oral intake only), 51.4% (drip infusion) and 55.6% (intensive methods) of each case. Among the 47 participants in the oral intake only group, 21 (44.7%) and 3 (6.4%) received DIV and PEG, respectively, during their end‐of‐life care.

**Table 3:** Preferences of AHN at the latest advance directives and actual status during their end of life

		Actual status of AHN in their EOL		
Preferences of AHN at the time of the latest ACP	PEG/NG tube/TPN	DIV	Oral	total
PEG/NG tube/TPN(*n*)	5	3	1	9
(%)	55.6	33.3	11.1	100.0
DIV (*n*)	5	19	13	43
(%)	13.5	51.4	35.1	100.0
Oral (*n*)	3	21	23	47
(%)	6.4	44.7	48.9	100.0
Total (*n*)	13	43	37	93
(%)	14.0	46.2	39.8	100.0

ACP, advance care planning; AHN, artificial hydration and nutrition; DIV, drip infusion through peripheral vein; EOL, end of life; NG, nasogastric; PEG, percutaneous endoscopic gastrostomy; TPN, total parenteral nutrition.

## Discussion

This study revealed that 60% of older people with AD preferred “oral intake only” over AHN during their end‐of‐life care. Many of the participants who died in the care home had received AHN, and those with AD received care according to their preferences. However, not all participants had their preferences respected. To the best of our knowledge, this is the first prospective study to clarify older adults' preferences for and actual status of AHN during end‐of‐life care in Japan. Older people are often faced with the need to make medical decisions near the end of life, when most of them lack the ability to do so.^21^ As many geriatricians experience ethical dilemmas and difficulty when introducing AHN to patients with end‐stage dementia, the Japanese Geriatric Society issued guidelines regarding decision‐making processes in elderly care, particularly about introducing AHN, in 2012.^12^ These are considered the first practical guidelines of ACP in Japan, although ACP has been recommended by the Japanese Ministry of Health, Labour, and Welfare since 2018.^22^ ACP discussions are particularly important for residents of care homes to make sure their wishes and preferences for end‐of‐life care are clear and implemented accordingly, but a specific order of medical care (e.g., implementation of AHN) is often lacking. Teno *et al*. reported that AD often fail to guide medical decision‐making; in the SUPPORT study, only 5.2% (*n* = 688) contained specific instructions regarding life‐sustaining treatments such as “No advanced life support, No ventilator, No NG tube.”^23^, ^24^ In the present study, in addition to preferred life‐sustaining treatments and place of death, participants were instructed to select their AHN preferences, so that their values could be respected during their end‐of‐life care. One definition of ACP is “understanding and sharing one's personal values, life goals, and preferences regarding future medical care,” and sometimes discussing preferences of AHN in the end of life.^9^, ^10^ In the present study, while AHN preferences might not have been sufficiently discussed during Wave 1 (i.e., AD only), in Wave 2, the advantages and drawbacks of each AHN method were explained to participants with illustrations during the ACP process, which could have affected participant preferences. During the 11‐year follow‐up, 90 participants completed AD twice, and of these, 15 (16.6%) changed their AHN preferences. As participants' values and preferences might change over time, AD should be updated regularly. Thus, a reminder for AD highlighting the importance of ACP conversations was sent to participants every year as a part of the annual CGA questionnaire. Moreover, levels of knowledge regarding AHN and health literacy may also vary among the older adults. In this regard, it is important to provide information on daily oral hygiene, swallowing training and food forms, as well as explanations about AHN. To our knowledge, the advantages and drawbacks of different AHN methods are rarely explained to older people living in care homes or in the community until their swallowing function deteriorates. This information should be provided during ACP conversations, as appropriate.

There are some limitations to this study. First, 27 (22.5%) of the 120 participants with AD died in hospitals, and their AHN statuses were unknown. It is likely that most participants who died in hospitals had been given DIV. Second, the reasons why older adults' preferences did not match the actual AHN provided at the end of life were unclear in most cases, with a few exceptions. Three participants in the oral intake only group received PEG during their end‐of‐life care; in one case, the participant's relatives did not participate in ACP conversations but strongly requested PEG tube placement, i.e., differences in values between the participant and the family was the reason for the mismatch. As for the 21 participants in the oral intake only group who received DIV, it is possible that their relatives or medical staff believed DIV to be an effective treatment option. Third, although AD documents including AHN preferences were available for all of the residents in the care home, it seemed difficult for residents with dementia to understand deeply the merit and demerit of each AHN method at the baseline. Thus, people with dementia at the baseline were excluded from the study. Fourth, our findings were from a single care home and may not be applicable to the general Japanese population.

In conclusion, most participants with AD received care according to their preferences. Public awareness of ACP regarding AHN should be raised not only from the perspective of respecting patient rights but also for the purpose of preventing ethical dilemmas for family caregivers and medical staff, as well as ensuring fair distribution of scarce care resources. To prepare for advanced dementia and senility, ACP conversations regarding AHN should be initiated early on.

## Disclosure statement

The authors declare no conflict of interest.

## Author contributions

TW was responsible for the design, conceptualization, methodology, acquisition of data, analysis and interpretation of data, and preparation of manuscript. YI, KH, EK, MT, MF, YM, YK, EF, HI, MN, MN, MI, SK, MH, and KO acquired the data, and were responsible for the writing (review and editing) of this paper. KM acquired the data, and was responsible for the writing (review and editing) and supervising of this study. RS wrote the paper (reviewed and edited) and performed project administration.

## Sponsor's role

None.

## Data Availability

The dataset generated during the current study are not publicly available due to privacy or ethical restrictions, but are available from the corresponding author on reasonable request.
